# Sleep habits, academic performance, and the adolescent brain structure

**DOI:** 10.1038/srep41678

**Published:** 2017-02-09

**Authors:** Anna S. Urrila, Eric Artiges, Jessica Massicotte, Ruben Miranda, Hélène Vulser, Pauline Bézivin-Frere, Winok Lapidaire, Hervé Lemaître, Jani Penttilä, Patricia J. Conrod, Hugh Garavan, Marie-Laure Paillère Martinot, Jean-Luc Martinot, The IMAGEN consortium, Tobias Banaschewski, Herta Flor, Mira Fauth-Bühler, Louise Poutska, Frauke Nees, Yvonne Grimmer, Maren Struve, Andeas Heinz, Andreas Ströhle, Viola Kappel, Betteke Maria van Noort, Jean-Baptiste Poline, Yanick Schwartz, Benjamin Thyreau, James Ireland, John Rogers, Nadège Bordas, Zuleima Bricaud, Irina Filippi, André Galinowski, Fanny Gollier-Briant, Vincent Ménard, Gunter Schumann, Sylvane Desrivières, Anna Cattrell, Robert Goodman, Argyris Stringaris, Charlotte Nymberg, Laurence Reed, Gareth J Barker, Berndt Ittermann, Ruediger Brühl, Michael Smolka, Thomas Hübner, Kathrin Müller, Arun L. W. Bokde, Christian Büchel, Uli Bromberg, Jurgen Gallinat, Tahmine Fadai, Pennylaire Gowland, C Lawrence, Tomas Paus

**Affiliations:** 1 Institut National de la Santé et de la Recherche Médicale, INSERM Unit 1000 “Neuroimaging & Psychiatry”, University Paris Sud – University Paris Saclay, University Paris Descartes, 97 Bd de Port Royal, 75014, Paris, France; 2 National Institute for Health and Welfare, Department of Health, Unit of Mental Health, P.O. Box 30, 00271 Helsinki, Finland; 3 University of Helsinki and Helsinki University Central Hospital, Department of Psychiatry/Adolescent Psychiatry, P.O. Box 803, 00029 HUS, Helsinki, Finland; 4 Department of Psychiatry 91G16, 4 place du Général Leclerc, Orsay Hospital, GH Nord-Essonne, 91400 Orsay, France; 5 Adolescent Psychiatry Department, Medical school, FI-33014 Tampere University, Finland; 6 Department of Psychiatry, Université de Montréal, CHU Ste Justine Hospital, 175 Chemin de la Côte-Sainte-Catherine, Montréal, QC H3T 1C4, Canada; 7 Department of Psychological Medicine and Psychiatry, Institute of Psychiatry, Psychology & Neuroscience, King’s College, 16 De Crespigny Park, London SE5 8AF, United Kingdom; 8 Departments of Psychiatry and Psychology, 6436 UHC, University of Vermont. 1 South Prospect Street, Burlington, VT 05401, USA; 9 APHP, Adolescent Psychopathology and Medicine department, Maison de Solenn, Cochin Hospital, 97 Bd de Port Royal, 75014 Paris, France; 10 CENIR at ICM institute, Centre de Neuroimagerie de Recherche Bâtiment ICM, 47-83 boulevard de l’Hôpital, 75651 Paris Cedex 13,ss France; 11 Central Institute of Mental Health, Mannheim, Germany; 12 Charité Hospital, Berlin, Germany; 13 Commissariat à l’Energie Atomique, Paris, France; 14 Delosis, Twickenham, Greater London, United Kingdom; 15 The Department of Neuroimaging, Institute of Psychiatry, Psychology & Neuroscience, King’s College London, United Kingdom; 16 Physikalisch-Technische Bundesanstalt (PTB), Berlin; Germany; 17 University of Dresden, Germany; 18 Trinity College Dublin, Ireland; 19 University of Hamburg, Germany; 20 University of Nottingham, United Kingdom; 21 University of Toronto, Canada

## Abstract

Here we report the first and most robust evidence about how sleep habits are associated with regional brain grey matter volumes and school grade average in early adolescence. Shorter time in bed during weekdays, and later weekend sleeping hours correlate with smaller brain grey matter volumes in frontal, anterior cingulate, and precuneus cortex regions. Poor school grade average associates with later weekend bedtime and smaller grey matter volumes in medial brain regions. The medial prefrontal - anterior cingulate cortex appears most tightly related to the adolescents’ variations in sleep habits, as its volume correlates inversely with both weekend bedtime and wake up time, and also with poor school performance. These findings suggest that sleep habits, notably during the weekends, have an alarming link with both the structure of the adolescent brain and school performance, and thus highlight the need for informed interventions.

Adolescents today sleep less and experience more daytime sleepiness symptoms as compared to previous generations^1^,^2^. During adolescence, sleep undergoes major changes: sleep duration and depth decrease, and sleep shifts towards evening hours. A tendency towards eveningness becomes evident during the adolescent years as a result of internal and external influences on brain mechanisms regulating sleep and circadian rhythm^3^. Staying up late combined with early morning awakenings for school easily lead to insufficient sleep and accumulation of sleep debt during the school week. Adolescents typically attempt to pay back their sleep debt during weekends, especially by sleeping in on weekend mornings^4^. Since the combination of delayed bedtimes and early school start times results in sleep debt for a large portion of the adolescent population, there is an ongoing public debate on how to arrange school starting times that would be suitable, applicable, and beneficial to adolescents’ health^5^,^6^.

Evidence from both epidemiological and experimental sleep restriction studies suggests that insufficient and/or late timing of sleep potentially has a large spectrum of negative effects on adolescents’ academic success, health, and safety^7^. Not surprisingly, both short sleep and late sleeping hours have been shown to correlate with poor school performance, possibly via a pathway involving reduced attention and increased daytime somnolence^8^,^9^,^10^. Cognitive processes supported by the networks associated with the prefrontal cortex, such as attention and executive functions, appear especially sensitive to sleep loss^11^,^12^.

Despite the detrimental effects of poor and inadequate sleep on adolescent academic success and health, evidence on the effects of long-term sleep habits on the developing adolescents’ brain structure is still lacking. The only published study of brain grey matter volumes (GMVs) in relation to sleep habits was performed in a mixed sample of children and adolescents. It showed that sleep duration during weekdays correlated with GMVs in bilateral hippocampal and right dorsolateral prefrontal cortex (DLPFC)^13^. Taking into account the radical changes in sleep structure, length, and timing during adolescent development, the need for studying a more homogeneous age group of adolescents appears evident. Furthermore, there are to date no reports on the associations between sleep timing and brain structure in this age group.

We examined the relationship between adolescents’ sleep habits and brain grey matter volume in a homogeneous sample of 14–year-old adolescents (n = 177) using magnetic resonance imaging (MRI) and voxel-based morphometry (VBM). The VBM approach gives a comprehensive assessment of anatomical volume differences throughout the brain without bias towards any specific region^14^. First, we hypothesized that shorter sleep, later bedtimes (indicating a tendency towards eveningness), and sleeping in on weekend mornings (indicating accumulation of sleep debt during the school week and recovery sleep during weekends) would be associated with smaller regional GMVs. Based on previous research^13^,^15^, we expected that the greatest changes in GMVs would be observed in the medial prefrontal and anterior cingulate cortex (ACC) as well as the hippocampus. Second, we hypothesized that sleep habits would correlate with school grade average, and, finally, that smaller GMVs correlating with sleep habits would be also associated with school grade average.

We found that, among adolescents, later weekend bedtimes correlated with smaller brain GMVs in frontal, anterior cingulate, and precuneus cortex regions. Later weekend bedtimes were associated with poorer school grade average, and both of these were further associated with small GMV in the medial PFC region. Shorter weekday time in bed correlated with smaller GMVs in frontal regions. These results highlight especially the possible adverse link of late timing of weekend sleep with the maturing adolescent brain and school performance.

## Results

Details on the sample are presented in Table 1.

### Sleep habits and GMVs

**Figure 1:**
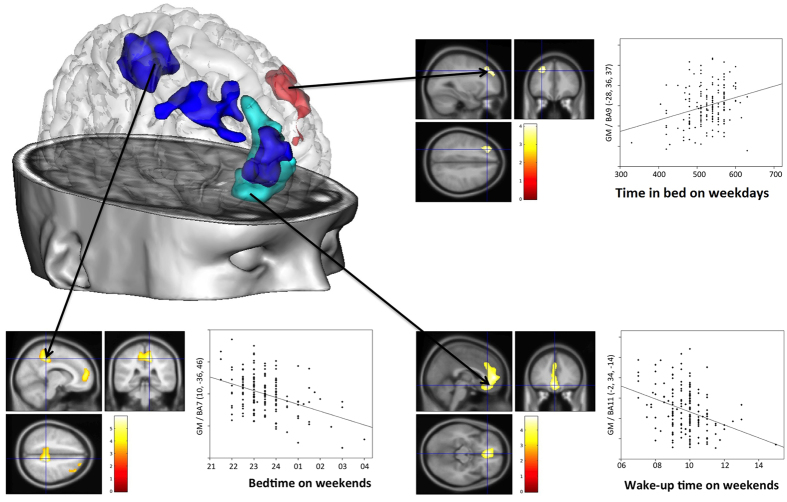
Brain regions where grey matter volumes correlated with sleep habits. Red colour and top right panel illustrate the DLPFC regions where GMV was correlated with time in bed on weekdays; light blue colour and bottom right panel illustrate the medial prefrontal and anterior cingulate regions where GMV correlated negatively with wake-up time on weekends; dark blue colour and bottom left panel illustrate the DLPFC, anterior cingulate and precuneus regions where GMV was negatively correlated with bedtime on weekends. All images are presented with height threshold p < 0.001, and FWE correction for multiple spatial comparisons across the whole brain (1200 voxels). The scatter plots are presented for the voxel of maximal statistical significance; the solid line represents the linear regression line. GMV is expressed in arbitrary units. BA = Brodmann area.

GMVs correlated with sleep habits in several cortical regions (Fig. 1, Table 2). Later wake up time on weekends correlated with smaller GMV in a cluster comprising the left frontal medial orbital cortex and the left ACC. Later bedtime on weekends correlated with smaller GMVs in three separate clusters located in (i) the right precuneus and paracentral lobule, (ii) the right middle/superior frontal gyrus, and (iii) the right frontal superior medial cortex and left ACC. Longer time in bed during weekdays correlated with larger GMV in a frontal cluster comprising the left superior and middle frontal gyrus. No other correlations between sleep habits and GMVs were observed.

### Sleep habits and school grade average

The average bedtimes, wake-up times, and time in bed on weekdays and on weekends are presented in Table 1. Boys had on average later bedtimes on weekends than girls (mean bedtime on weekends boys 23:40 ± 1:08 vs. girls 23:18 ± 00:59; independent samples t-test *p* = 0.020, t = −2.354). No other statistically significant gender differences were noted in sleep habits.

In regression analyses controlling for gender (β = 0.570), pubertal stage (β = −0.211), and IQ (β = −0.061), there was a relationship between poor school grade average and late weekend bedtimes (*p* = 0.00472; β = 0.211; t = 2.863). School grade average was not correlated with any other sleep habit.

### School grade average and GMV correlates

Based on the six voxel-wise correlation analyses between sleep habits and GMV, three *region*-*of interest* masks were identified for the following variables: (1) wake-up time during weekends (including areas of the left frontal medial orbital cortex and the left ACC); (2) bedtime during weekends (including areas of the right precuneus and paracentral lobule, the right middle and superior frontal gyrus, the right frontal superior medial cortex, and the left ACC); and (3) time in bed during weekdays (including areas of the left middle and superior frontal gyrus).

School grade average correlated with GMV within two of the three applied *region*-*of*-*interest* masks in medial brain regions: (i) within the weekend wake-up time *region*-*of*-*interest* mask, in a cluster of the bilateral anterior cingulate cortex extending to the bilateral frontal superior medial cortices, and (ii) within the weekend bedtime *region*-*of*-*interest* mask, in three clusters located in the left paracentral lobule and middle cingulate, the left anterior cingulate, and the right anterior cingulate cortex (Table 3). No statistically significant correlations between school grade average and GMV were found within the weekday time in bed *region*-*of*-*interest* mask.

Causal mediation analyses showed that weekend bedtime explained a significant fraction of the relationship between GMV and school performance (Table 4). Weekend bedtime mediated the relationship between the GMVs within two of the three *regions*-*of*-*interest* and school grade average. Weekend bedtime accounted for 43.8% (p < 0.001) of the total effect between GMV in the frontal superior medial cortex and ACC, and school performance. Also, it accounted for 33.7% (p = 0.03) of the total effect between GMV in the precuneus and paracentral lobule, and school performance. No mediation effect was found in any of the other mediation analyses performed.

## Discussion

Shorter weekday sleep and later weekend sleep habits associated with smaller brain regional GMV among 14-year-old community adolescents. The medial prefrontal cortex (PFC) GMV correlated both with bedtime and wake-up times during weekends, and it was also associated with school grade average. These findings implicate that the medial PFC, especially the anterior cingulate cortex, might be the brain region that is most sensitive to individual differences in sleep habits during adolescence.

Weekend bedtimes, weekend wake-up times, and weekday time in bed, which likely represent three separate but partly overlapping domains of sleep (as denoted in Supplementary Table S1), had differential correlations with brain regional GMV. While weekday time in bed describes the habitual sleep amount, weekend bedtime reflects a tendency towards eveningness, and weekend wake-up time possibly reflects recovery sleep due to accumulation of sleep debt during the week^3^,^4^. Herein, wake-up time and bedtime during weekdays and time in bed during weekends were not correlated with brain GMV, which is likely explained by the fact that school starting times dictate sleeping times, particularly wake-up times, during weekdays. Thus, inter-individual differences in sleep timing become more evident on weekends with a free schedule, while sleep curtailment occurs most commonly during weekdays^3^,^4^.

The most significant results were indeed related to timing of sleep during weekends: the later the adolescents slept during weekends, the smaller were their GMVs in a large cluster centered in the medial orbitofrontal cortex (OFC) and the anterior cingulate cortex (ACC). These medial PFC regions have been previously implicated in sleep pathologies^16^,^17^,^18^,^19^. Moreover, these regions have been observed in healthy adults to correlate positively with habitual ‘sleep credit’ (sleeping in excess of sleep need)^20^ and negatively with subjective sleepiness^21^. Our data adds to converging evidence that GMV of the ACC and medial OFC are related to sleep habits.

The PFC is among the latest maturing brain regions, with changes seen until the second decade of life^22^, which potentially makes it vulnerable to the effects of sleep habits during adolescence. At the age of 14 years, the peak GMV has already passed in frontal areas, and brain maturation is reflected in GMV reduction^23^. The medial PFC is involved in cognitive and emotional control, and selective attention^24^, and its function has been shown to be impaired during sleep deprivation^15^.

Later bedtime during weekends was additionally associated with widespread GMV reductions in the right middle, medial, and superior frontal cortex, and in the precuneus. Sleep slow wave activity, an electrophysiological measure of ‘deep’ sleep or sleep homeostasis, has been shown to be closely correlated with GM thickness in most of these brain regions in children and adolescents^25^. Herein, the middle and superior frontal gyri GMV correlated negatively with both weekend bedtime and weekday time in bed. They are part of the dorsolateral prefrontal cortex (DLPFC)^26^, thus extending previous findings linking DLPFC structure and function with sleep^13^, sleep loss^27^, and sleep pathologies^19^. The precuneus is an epicenter of the default mode network^28^. Its activity, similarly to the activity of the ACC, is highly variable during the healthy sleep-wake cycle: high during wakefulness and low during slow-wave sleep^29^. A reduction in precuneus GMV has been observed in insomnia patients^17^, but our study is the first to demonstrate an association between GMV of the precuneus and sleep habits in healthy individuals, using a whole brain VBM approach.

Poorer school grades associated with smaller GMV in the medial frontal areas. This is the first report to show that both sleep habits and school grades are linked to brain GM morphometry of the medial PFC in adolescents. Interestingly, these regions are epicenters for functions such as multitasking^30^ and self-related mental representations^28^ that might be at stake in adolescents’ academic achievements. Although the direction of the mediation analyses (i.e. that weekend bedtime mediated the relation between GMV and school performance) was not exactly as we initially hypothesized (that GMVs would mediate the effect of sleep habits on school performance), the results of the mediation analyses altogether suggest that late weekend bedtime might draw a deleterious relation between GMV and school grades. Based on our data it remains speculative how much e.g. modifying bedtimes could affect school performance. Further intervention studies are required to answer these questions.

Contrary to our expectations and previous findings^13^, we did not see a correlation of sleep habits with hippocampal GMV. This discrepancy may be attributed to methodological differences, notably the region-of-interest approach used in the study by Taki *et al*.^13^ as compared to the present whole-brain statistical parametric mapping approach, and the fact that maturation of the hippocampus follows a differential trajectory than the neocortex. Further, contrary to the volume of the neocortex, hippocampal volumes might even increase over the course of adolescence^31^. The hippocampus region might therefore be more vulnerable to sleep habits at a different phase of development than the neocortex, which could partly explain the differential findings between the present homogeneous age group of adolescents (age range 13.4–15.5 years) and the earlier study including a mixed sample of children and adolescents aged 5–18 years^13^. Finally, in the present VBM study we used a conservative extent threshold (1200 voxels), which may have precluded the detection in small brain structures. Indeed, in supplementary analyses with a more liberal threshold (p < 0.001 uncorrected; extent threshold at 50 voxels uncorrected), we found a correlation between smaller bilateral hippocampal volumes and later weekend bedtimes as well as shorter weekday time in bed (see Supplementary Figures S1 and S2). These correlations were, however, statistically much weaker than the neocortical findings.

The major strength of the current study is the sample with a narrow age range recruited from the general population. Additionally, the variability in pubertal developmental stage within the sample was taken into account by including the adolescents’ PDS scores as a confounding covariate in the statistical models. Thus the effects of age/pubertal status on our results were minimized. Moreover, the bedtimes and wake up times in our sample are in line with those previously reported in larger-scale epidemiologic studies on adolescent sleep habits^4^.

Although our sample included also a small proportion of individuals with probable psychiatric disorders, the main results were not accounted for by the presence of psychiatric morbidities among the participants (see Supplementary Table S2), implicating an independent role for sleep habits.

Limitations include the lack of objective measures of sleep and the cross-sectional design. Since no objective assessment of sleep was performed, the design does not allow any inferences on the more exact neurobiological mechanisms underlying the relationship between sleep habits and brain grey matter volumes.

Cross-sectional data cannot test for causal relationships between variables, i.e. whether poor sleep habits are causing or caused by regional GMV loss. The cross-sectional design of the study also precludes inferences related to brain maturation. Longitudinal approaches are needed to address whether the GMV changes are in fact harmful or helpful for the individuals in the long run. During adolescent years the normative course of development is towards a decrease in both GMVs and sleep duration, and towards delayed sleep timing: the more mature the adolescent is, the less GMV(s) he is expected to have^3^,^23^. The sample we studied was particularly homogeneous in terms of age, and, furthermore, pubertal developmental stage was controlled in the analyses. On a cautious note, we would not expect that the findings would reflect a positive maturational change, but rather a deviation in maturation, i.e. a GMV decrease beyond the average levels. The link between smaller GMV and poorer school performance would also favor the idea of the changes to be rather maladaptive than developmentally favorable. The literature on sleep pathologies in adult patients would also support the second alternative.

Finally, the determinants of adolescents’ sleep habits involve biological, behavioural, psychosocial, and cultural factors that are difficult to disentangle, and may interfere with brain maturation. Demonstrating the role of these factors was beyond our objectives.

In conclusion, the present report highlights an alarming association between the variations in sleep habits, adolescent regional GMV, and school grade average. Irrespective of the causal underpinnings of this relationship, the findings support that paying attention to sleep habits during this period of maturation might be a precautionary principle. We would encourage all parental, societal, and educational support for adolescents to ensure maintenance of a healthy sleep-wake rhythm. Especially, avoiding late bedtimes during weekends would seem important in order to make the most out of the brain’s developmental potential and to ensure optimal academic success.

## Methods

### Participants

A total of 265 adolescents were recruited based on their age from the general population from 9^th^ and 10^th^ grade students in schools near Paris, France, via flyers and school visits. Exclusion criteria included severe medical somatic conditions, any history of head injuries, and any contraindications for MRI. These adolescents participated in a larger European multi-site longitudinal study of adolescent development^32^. Only the French adolescents were assessed for their sleep characteristics and were thus eligible for the study. For the purpose of this study, we excluded all participants who dropped out before the MRI scan, participants with missing sleep data, or whose brain images did not pass quality control, or with marked alcohol consumption (Alcohol Use Disorders Identification Test (AUDIT) total score > 7), resulting in the exclusion of 88 adolescents. Here, we thus present data from the remaining 177 adolescents.

The study protocol was approved by the Ethics Committee of Paris CPP IDF-VII and the study was conducted in accordance with the Declaration of Helsinki. Written informed assent and consent were obtained from all the participants and their parents, respectively.

The participants were assessed for psychiatric symptoms with the Development and Well-Being Assessment (DAWBA) (), a self-administered computer-based diagnostic questionnaire consisting of open and closed questions^33^. Other assessments included the Pubertal Development Scale (PDS) questionnaire^34^, and the Wechsler Intelligence Scale for Children-Fourth Edition (WISC-IV)^35^. According to DAWBA, the majority of the adolescents were free of any clinically significant psychiatric symptoms, while n = 28 (15.8%) adolescents in total were rated as having a probable DSM-IV psychiatric diagnosis. Although some participants expressed psychiatric symptoms, none of them was using psychotropic medication or mental health consultation at the time of the study, and all participants were attending school regularly.

Supplementary analyses on the regional correlations between sleep habits and GMVs were also performed in a subsample of 149 adolescents without any probable psychiatric diagnosis (Supplementary Table S2).

#### Assessment of sleep

Sleep habits were assessed by asking the adolescents their habitual bedtimes and wake-up times during weekdays and weekends separately. The exact questions were “Average time of going to bed on weekdays”; “Average time of waking up during the week”; “Average time of going to bed during the weekend”; “Average time of waking up during the weekend” (original questions in French: “Heure moyenne de coucher la semaine, Heure moyenne de lever la semaine; Heure moyenne de coucher le week-end; Heure moyenne de lever le week-end”). The questions did not concern a specific retrospective time range but the adolescents were asked to report their usual sleeping habits. Time in bed was calculated based on the bedtimes and wake-up times reported by the participants separately for weekdays and weekends. The sleep assessments were performed on the same day as the MRI.

### Assessment of school grade average

The participants reported their school grade average of the last term as part of the European School Survey Project on Alcohol and Drugs (ESPAD)^36^ by answering the question ”Which of the following best describes your average grade in the end of the last term”? with an 8-point scale ranging from A (93–100) to C- (70–72). (See Supplementary Table S3 for details).

### MRI data acquisition and preprocessing

All adolescents underwent MRI examination with a Siemens Trio 3 Tesla scanner at the Neurospin Centre (France). High-resolution structural T1-weighted images were obtained using a standardized 3D T1-weighted magnetization prepared rapid acquisition echo (MPRAGE) sequence based on the ADNI protocol (http://adni.loni.usc.edu/methods/mri-analysis/mri-acquisition/) with the following acquisition parameters: repetition time = 2300 ms: echo time = 2.8 ms, flip angle = 8°; 256 × 256 × 170 matrix, 1.1 × 1.1 × 1.1 mm voxel size). Images were preprocessed with Statistical Parametric Mapping 8 software (SPM8) using Voxel-Based Morphometry (VBM)^7^. The T1-weighted images were segmented and normalized using customized Tissue Probability Maps. The normalized, segmented and modulated grey matter (GM) images were smoothed using a 10-mm full-width at half-maximum (FWHM) Gaussian kernel. Head size was measured by the Volume Scaling Factor (VSF), which is based on the affine transformation performed during spatial normalization (https://surfer.nmr.mgh.harvard.edu/fswiki/eTIV).

### Statistical analysis

The demographic, sleep and school performance variables were analysed with the IBM SPSS Statistics Version 22. Regression analyses using the general linear model (GLM) method were conducted to assess whether sleep habits were associated with school grade average. These analyses were controlled for pubertal stage (PDS) and gender. Bonferroni-corrected p-values of <0.0083 were considered as statistically significant.

The correlations between sleep habits - bedtime, wake up time, and time in bed, separately on weekdays and weekends - and GMVs, were examined in the *whole brain* using multiple regression models in SPM8. For each of the six correlation analyses, we used one sleep habit as covariate of interest, and gender, PDS and VSF as confounding covariates. Significant clusters of GMVs were identified using a height threshold of p < 0.001, with family-wise error (FWE) correction for multiple spatial comparisons at the cluster level across the whole brain (p < 0.05, spatial extent 1200 voxels) under non-stationarity assumption^37^. Afterwards, analyses were conducted in order to determine whether the regions where GMVs correlated with sleep habits did also relate with school grade average. These analyses were conducted within 3 *region*-*of*-*interest masks* obtained from significant correlation analyses between sleep habits and GMVs. Correlation analyses between the school grade average and GMV within the *region*-*of*-*interest* masks were conducted with a height threshold set at p < 0.05 FWE corrected for multiple comparisons.

Causal mediation analyses were conducted to determine whether, in the *regions*-*of*-*interest* that were found to correlate with sleep habits, (1) sleep habits could mediate the relation between the GMVs and school grade average, or (2) GMVs could mediate the relation between the sleep habits and school grade average. Causal mediation analyses were performed with algorithms devised by Imai *et al*.^38^. School grade average was entered as dependent factor and (1) GMVs and (2) sleep habits (wake up time and bedtime during weekends) as independent factor. (1) Sleep habits and (2) GMVs were entered as mediator variable. Gender, PDS and VSF were entered as confounding variables. The mediator models were fit with the general linear model and output objects were bootstrapped 5000 times with replacement using a parametric mediational analysis. In causal mediation analyses, a significant mediating effect was defined as a 95% confidence interval that does not include zero.

## Additional Information

**How to cite this article:** Urrila, A. S. *et al*. Sleep habits, academic performance, and the adolescent brain structure. *Sci. Rep.* **7**, 41678; doi: 10.1038/srep41678 (2017).

**Publisher's note:** Springer Nature remains neutral with regard to jurisdictional claims in published maps and institutional affiliations.

## Supplementary Material

Supplementary Information

## Figures and Tables

**Table 1:** Characteristics and brain volumes in 177 community adolescents.

Variable		Mean (or %)^a^	SD
Demography	Age	14.4	0.5
	Gender	52.0% (n = 92) female	
	BMI	20.1	2.7
	IQ	109.3	9.0
	PDS	2.9	0.6
Sleep habits	Wake up time WD	7:06	0:23
	Wake up time WE	9:45	1:09
	Bed time WD	22:20	0:44
	Bed time WE	23:29	1:05
	Time in bed WD (h)	8.8	0.8
	Time in bed WE (h)	10.3	1.2
School performance	Grade average	2.54	1.22
Global brain volumes	Total GMV	805.2	77.7
	Total WMV	452.8	48.8
	Total CSF	541.5	95.6
	Total volume	1799.49	178.12

^a^% instead of mean is indicated for nominal variables when appropriate. BMI = body mass index; IQ = intelligence quotient; PDS = Pubertal Development Scale score; WD = weekday; WE = weekend; DAWBA = Development and Well-Being Assessment; GMV = grey matter volume; WMV = white matter volume; CSF = cerebrospinal fluid.

**Table 2:** Regional correlations between sleep habits and grey matter volumes in community adolescents.

Brain region	Cluster			Peak voxel					
	BA	k	p	MNI coordinates			p^a^		t
				x	y	z			
***Time in bed during weekdays***									
Superior frontal gyrus L	9	1222	0.049	−28	36	37	3.41E-05		4.08
Middle frontal gyrus L	8			−39	30	46	5.04E-05		3.98
***Wake up time during weekends***									
Frontal medial orbital L	11	5163	5.68E-05	−2	34	−14	1.35E-06	*	4.85
Anterior cingulate L	32			−2	50	4	1.84E-06	*	4.78
***Bedtime during weekends***									
Precuneus R	7	3068	0.0014	10	−36	46	7.86E-09	*	5.94
Paracentral lobule L	5			−12	−33	52	7.31E-06	4.46	
Middle frontal gyrus R	9/10	2220	0.0062	33	27	33	5.23E-07	*	5.06
Superior frontal gyrus R	8			38	30	49	1.15E-04		3.76
Frontal superior medial R	10	3219	0.0011	14	56	10	5.24E-07	*	5.06
Anterior cingulate L	32/24			−3	44	−5	6.88E-05		3.90

BA = Brodmann Area; k = cluster size, expressed in number of voxels; MNI = Montreal neurological Institute coordinates in millimeters; R = right; L = left. MNI coordinates are given for the voxel of maximal statistical significance. Correlations are negative for wake up time and bedtime during weekends, and positive for time in bedduring weekdays.

^a^Height threshold p < 0.001; cluster extent threshold p < 0.05 FWE corrected (k > 1200 voxels). *Height threshold p < 0.05 Family Wise Error (FWE) corrected.

**Table 3:** Regional negative correlations between school grade average and grey matter volumes in community adolescents.

Brain region	BA	Cluster		Peak voxel				
		k	p	MNI coordinates			p^a^	t
				x	y	z		
***region***-***of***-***interest mask**: **wake up time weekend***								
Anterior cingulate L	32	739	0.0004	−10	46	10	1.34E-05	4.32
Frontal superior medial L	32			−3	27	36	1.41E-04	3.74
Anterior cingulate L	24			−2	33	0	2.03E-04	3.54
Anterior cingulate R	32			4	39	−2	2.29E-04	3.50
Frontal superior medial R	10			2	52	15	4.71E-04	3.27
***region***-***of***-***interest mask**: **bedtime weekend***								
Mid Cingulate L/Paracentral lobule	31	183	0.011	−12	−30	51	4.18E-05	4.03
Mid Cingulate R	31			4	−27	46	5.33E-05	3.97
Anterior cingulate L	32	122	0.016	−4	45	3	6.63E-05	3.91
Anterior cingulate L	24			−2	33	0	2.03E-04	3.62
Anterior cingulate R	32	7	0.042	6	39	−2	1.80E-04	3.62

^a^Height threshold p < 0.05 FWE corrected for multiple comparisons, extent threshold p < 0.05 FWE corrected. BA = Brodmann Area; k = number of voxels; MNI = Montreal neurological Institute coordinates in millimeters; R = right; L = left. MNI coordinates are given for the voxel of maximal statistical significance.

**Table 4:** Causal mediation analyses on the relationship between the GMVs and school grade average with bedtime during weekends as mediator.

Effect type	Point estimate	95% CI	p-value
***Precuneus***/***paracentral cluster** (**BA 7***/***BA 5***)			
**Mediation effect**	−1.429	[−2.948; −0.163]	0.03
(GMV – bedtime – school performance)			
**Direct effect**	−2.765	[−5.825; 0.322]	0.08
(GMV – school performance)			
**Total effect**	−4.194	[−7.052; −1.340]	<0.001
**Proportion of total effect via mediation**	0.337	[0.030; 1.158]	0.04
***Frontal superior medial and ACC cluster** (**BA10***/***BA 32***)			
**Mediation effect**	−2.339	[−4.807; −0.562]	<0.001
(GMV – bedtime – school performance)			
**Direct effect**	−2.851	[−7.764; 1.978]	0.28
(GMV – school performance)			
**Total effect**	−5.190	[−9.821; −0.884]	0.02
**Proportion of total effect via mediation**	0.438	[0.075; 2.191]	0.02

GMV = Grey matter volume; BA = Brodmann Area; 95% CI = 95% confidence interval of the point estimate.
