# Supplementary material for: Sleep habits, academic performance, and the adolescent brain structure
Source: Sci Rep. 2017 Feb 9;7:41678. doi: 10.1038/srep41678 (PMC5299428; doi:10.1038/srep41678)

Sleep habits, academic performance, and the adolescent brain structure

Anna S. Urrila, Eric Artiges, Jessica Massicotte, Ruben Miranda, Hélène Vulser, Pauline Bézivin-Frere, Winok Lapidaire, Hervé Lemaître, Jani Penttilä, Patricia J Conrod, Hugh Garavan, the IMAGEN consortium, Marie-Laure Paillère Martinot, and Jean-Luc Martinot

Supplemental information

Discussion

Supplementary table S1

Spearman rank correlation coefficients for sleep habits and participant characteristics.

TIB WE	TIB WD	Wake up time WE	Wake up time WD	Bedtime WE	Bedtime WD	School grade average	IQ	PDS	Gender	
									1	**Gender**
								1	-.549**	**PDS**
							1	-.110	.077	**IQ**
						1	-.411**	-.175	.273**	**School grade average**
					1	.121	-.052	.134	.054	**Bedtime WD**
				1	.659**	.261**	-.084	.045	.186*	**bedtime WE**
			1	.008	.058	.052	-.052	-.137	.086	**wake up time WD**
		1	.098	.405**	.321**	.131	-.201**	.005	.073	**wake up time WE**
	1	-.204**	.374**	-.532**	-.839**	-.046	.003	-.199**	.009	**TIB WD**
1	.251**	.521**	.097	-.445**	-.261**	-.055	-.115	-.058	-.092	**TIB WE**

Significant correlations are marked with *at the $p<0.05$ level and **at the $p<0.01$ level. PDS = pubertal; Development Scale; IQ = intelligence quotient; WD = weekdays; WE = weekends; TIB = time in bed. Gender is described as 1 = girl; 2 = boy; school grade average is described on a scale from 1 to 8 where 1 = very good, 8 = very poor.

Supplementary table S2. Regional correlations between sleep habits and grey matter volumes in a subsample of 149 community adolescents without any probable psychiatric diagnosis according to the DAWBA questionnaire.

		Cluster		Peak voxel					
Brain region	BA	k	p	MNI coordinates			p^a		t
				x	y	z			
Wake up time during weekends									
Anterior cingulate L	32	3327	8.90E-04	-3	36	-11	7.37E-06		4.49
Frontal medial orbital L	32			0	33	31	2.22E-04		3.60
Bedtime during weekends									
Precuneus R	7	1668	0.018	9	-36	34	9.28E-04	*	4.97
Paracentral lobule L	5			-12	-33	52	7.81E-05		3.88
Middle frontal gyrus R	8/9/10	2299	0.0053	34	30	34	8.31E-08	*	5.50
Parahippocampal R	20	1659	0.019	38	-21	-23	4.33E-05		4.04
Inferior temporal R	20/36			48	-37	-18	5.01E-05		4.00

a. Height threshold p<0.001; cluster extent threshold p<0.05 FWE corrected (k>1200 voxels). * Height threshold p<0.05 Family Wise Error (FWE) corrected. BA = Brodmann Area; k = cluster size; MNI = Montreal neurological Institute coordinates in millimeters; R = right; L = left. MNI coordinates are given for the voxel of maximal statistical significance.

Methods

Supplementary table S3. Assessment of school grade average; correspondence of the English and French questionnaires.

language	Question	Response scale
English (UK)	'Which of the following best describes your average grade in the end of the last term?'	1) A (93-100)<br>2) A- (90-92)<br>3) B+ (87-89)<br>4) B (83-86)<br>5) B- (80-82)<br>6) C+ (77-79)<br>7) C (73-76)<br>8) C- (70-72)
French	'Quelle a été ta moyenne générale (approximativement) à la fin du dernier trimestre?'	1) 16-20<br>2) 14-15<br>3) 12-13<br>4) 10-11<br>5) 8-9<br>6) 6-7<br>7) 4-5<br>8) 0-3

Supplementary figure S1. Bilateral correlations between bedtime during weekends and GMV in the hippocampal regions. Analyses performed with a height threshold p=0.001 and an extent threshold = 50 voxels. Crosshair indicates left hippocampus at MNI coordinates x, y, z: -26, -21, -20 (t = 3.73; p = 1.28 E-04); right parahippocampus is at MNI coordinates x, y, z: 37, -19, -21 (t = 3.97; p = 5.17 E-05).

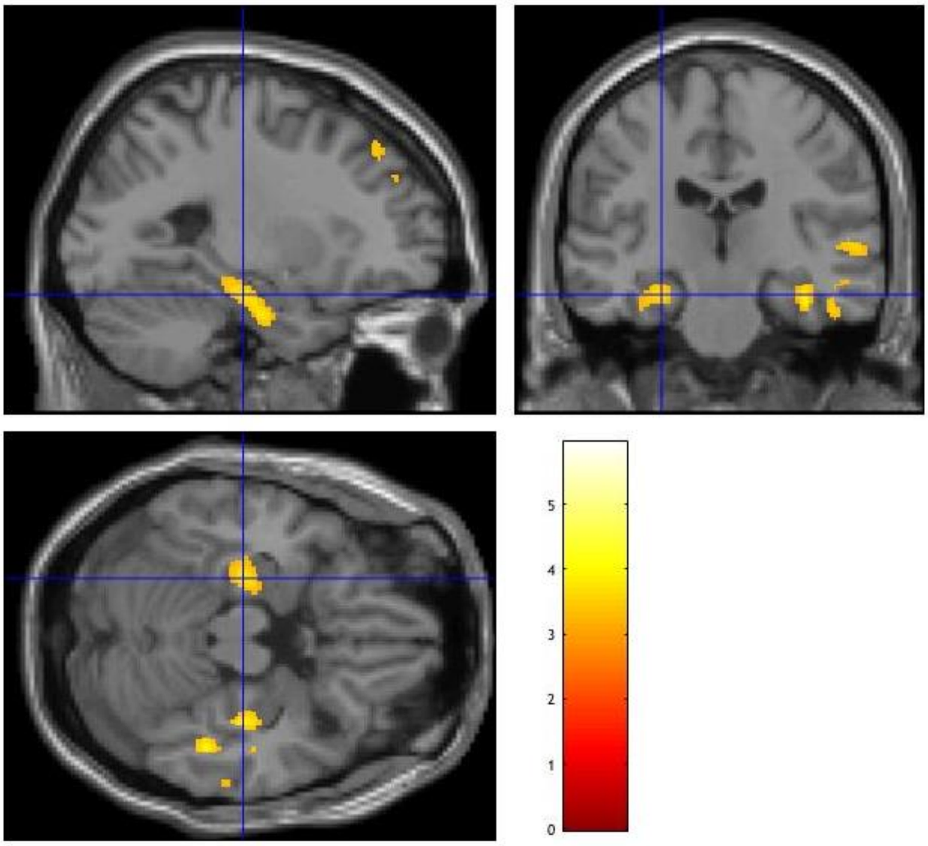

Supplementary figure S2. Bilateral correlations between weekday time in bed and GMV in the hippocampal regions. Analyses performed with a height threshold p=0.001 and an extent threshold = 50 voxels. Crosshair indicates left hippocampus at MNI coordinates x, y, z: -27, -21, -20 (t = 3.29; p = 5.99 E-04); right parahippocampus is at MNI coordinates x, y, z: 38, -22, -21, (t = 3.61; p = 2.04 E-04).

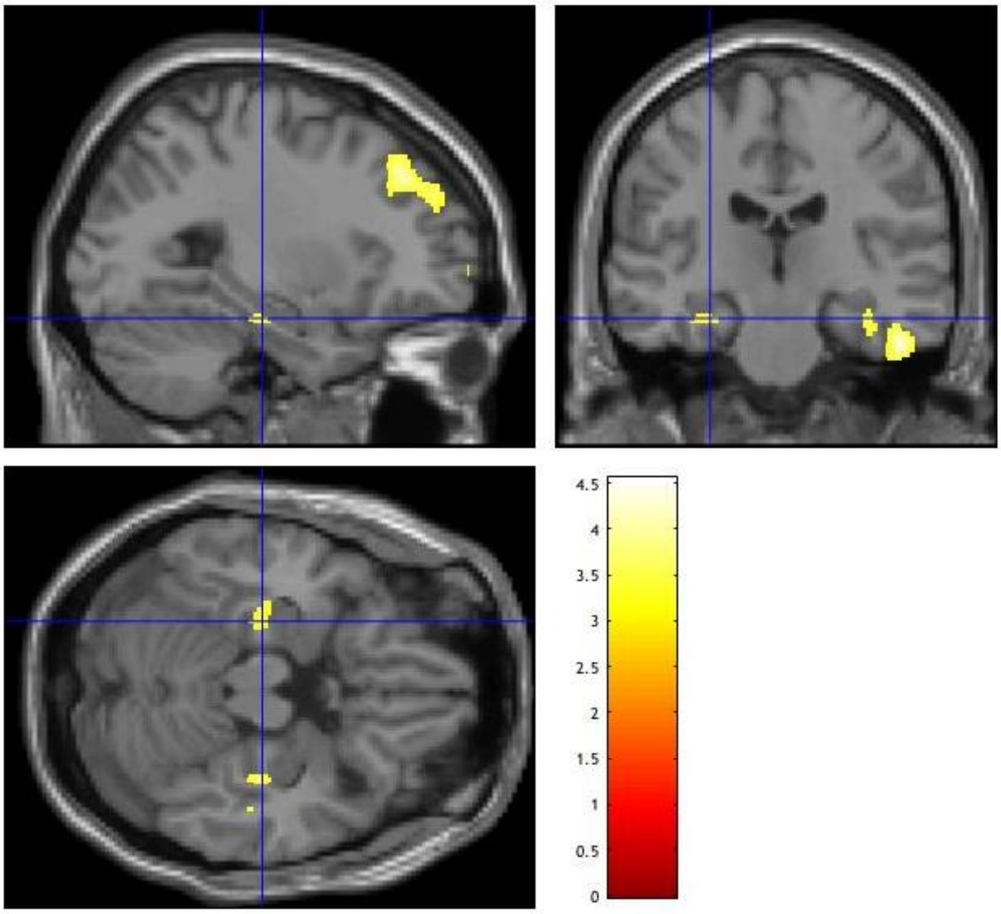

Supplement: Supplementary Information [file srep41678-s1.pdf]
